# Supplementary material for: Structure of the RBM7–ZCCHC8 core of the NEXT complex reveals connections to splicing factors
Source: Nat Commun. 2016 Dec 1;7:13573. doi: 10.1038/ncomms13573 (PMC5146272; doi:10.1038/ncomms13573)
Supplement: Supplementary Information — Supplementary Figures 1 - 5 and Supplementary References [file ncomms13573-s1.pdf]

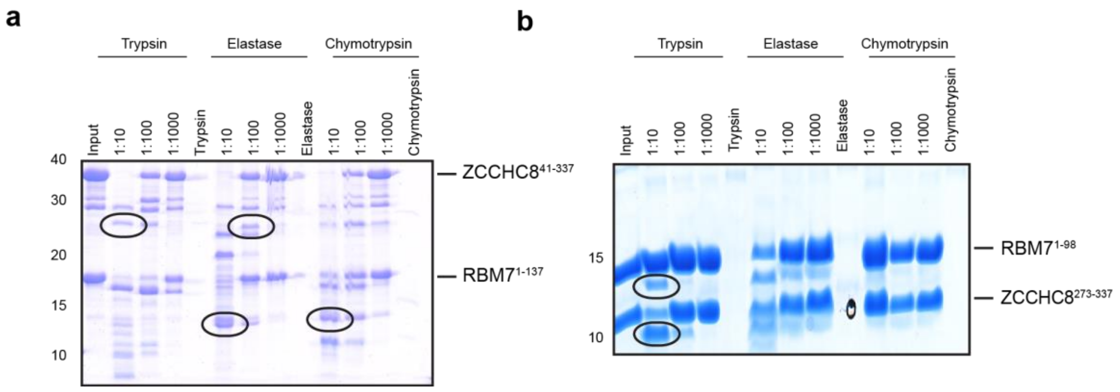

Supplementary Figure 1 Limited proteolysis of RBM7 – ZCCHC8 complexes

(**a**) Complex of ZCCHC8$^{41\text{-}337}$ and RBM7^{137} was incubated for 30 minutes on ice with trypsin, elastase and chymotrypsin at the indicated ratios. The proteolytic fragments were separated on a Coommassie-stained SDS-PAGE gel. From mass spectrometry analyses (data not shown), the encircled bands were identified to correspond to an N-terminally truncated ZCCHC8 product and C-terminally truncated RBM7 product. (**b**) Complex of ZCCHC8$^{273\text{-}337}$ and RBM7$^{1\text{-}98}$ was incubated with trypsin, elastase or chymotrypsin as in (a). From mass spectrometry analyses (data not shown), the encircled bands were identified to correspond to ZCCHC8$^{285\text{-}325}$ and RBM7$^{1\text{-}86}$.

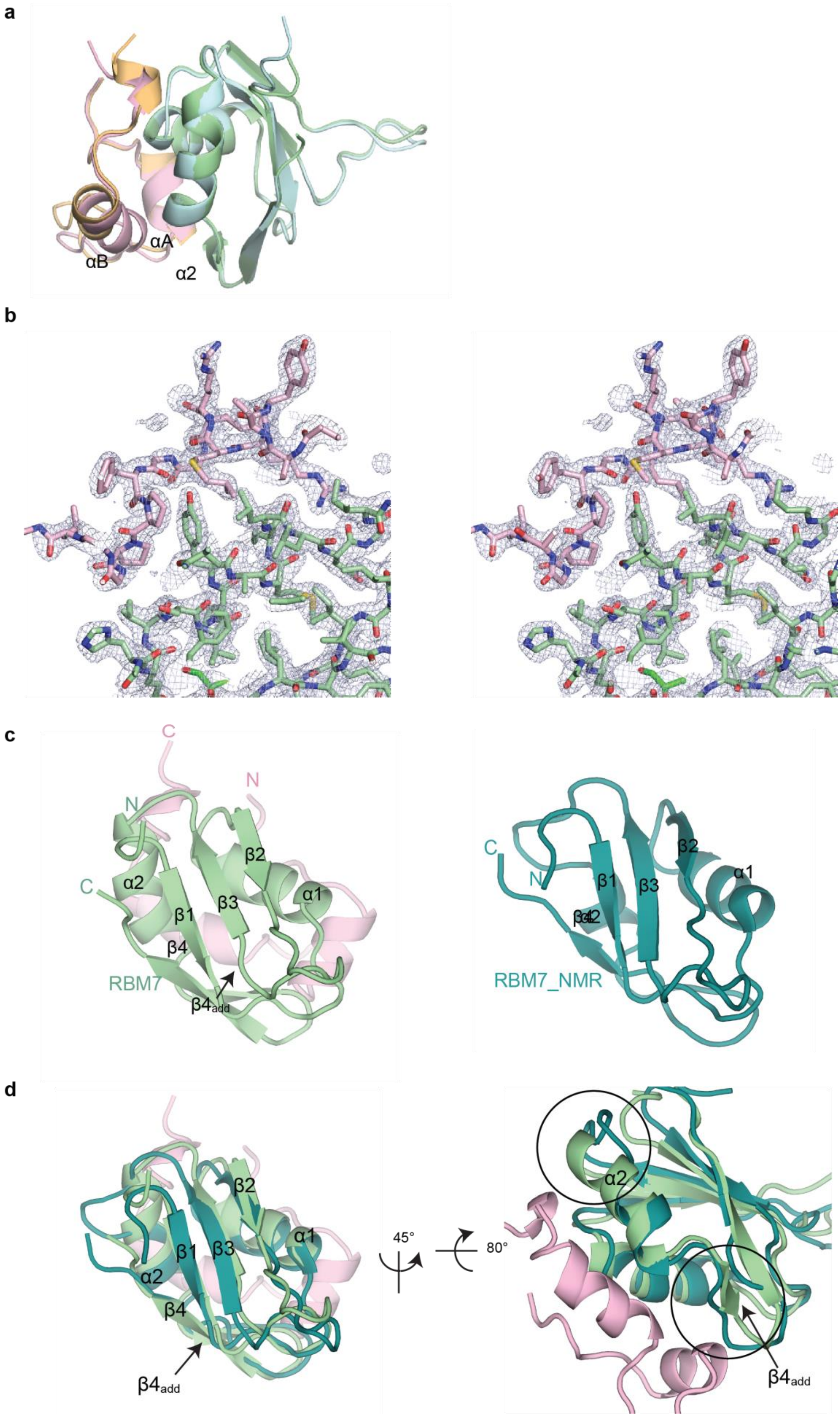
a
αA
αB
α2
b
c
C
N
N
C
α2
β2
β3
β1
α1
β4
RBM7
$\beta4_{add}$
C
N
β1
β3
β2
α1
RBM7_NMR
d
β2
β1
β3
α1
α2
β4
$\beta4_{add}$
45°
80°
α2
$\beta4_{add}$

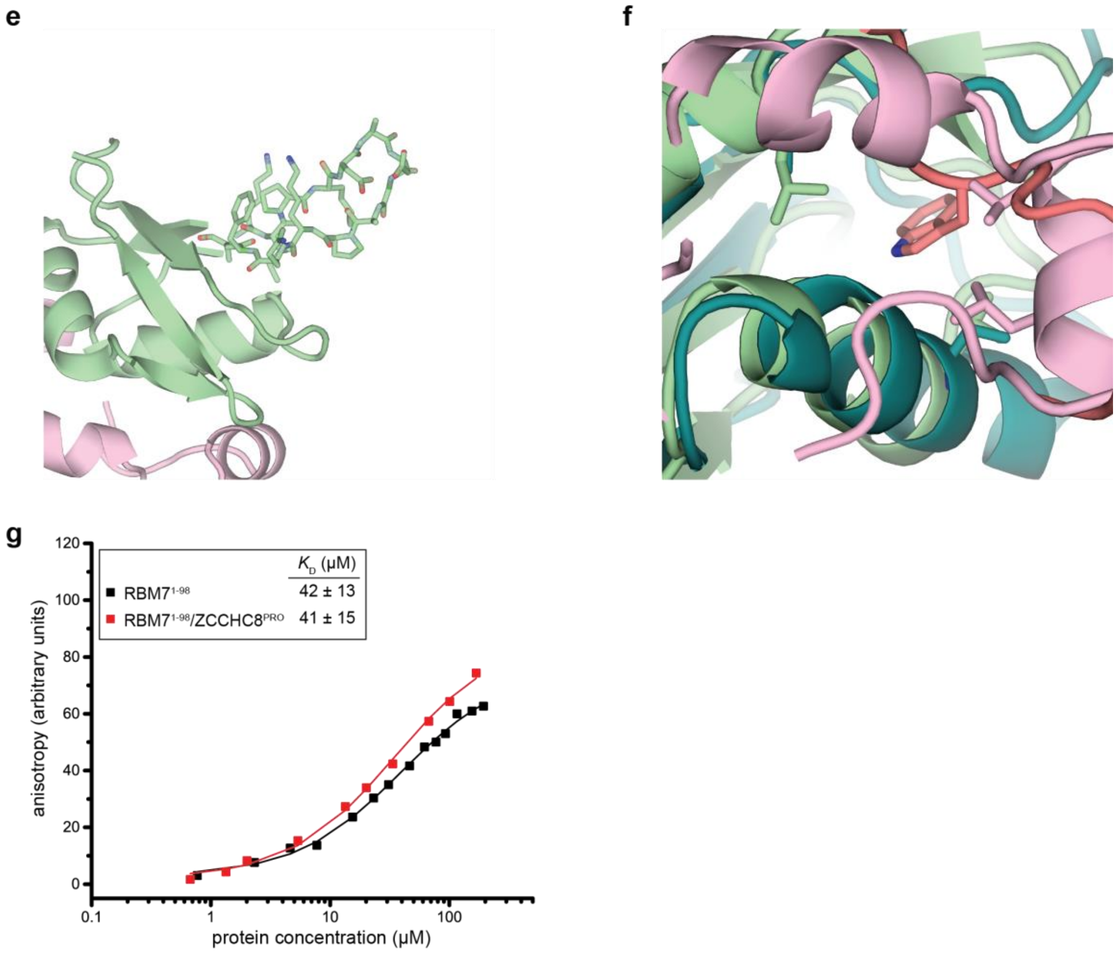

Supplementary Figure 2 Analysis of the RBM7^{RRM}-ZCCHC8^{Pro} structure

(**a**) Comparison of the RBM7 – ZCCHC8 structures obtained from the native and Samarium derivatized crystals. Superposition of one copy (Chain A and B) from the native RBM7 – ZCCHC8 (light blue and orange) structure onto the Samarium-derivatized RBM7-ZCCHC8 structure (green and pink). (**b**) Stereo image of the electron density 2mFo-DFc map (grey mesh) contoured at 1.5 σ near the RBM7^{RRM}-ZCCHC8^{Pro} interface. The representation is colored by element, with RBM7^{RRM} carbon atoms colored in green and ZCCHC8^{Pro} carbon atoms colored in pink. (**c**) and (**d**) Comparison of the RBM7 – ZCCHC8 crystal structure with the RBM7 NMR structure (PDB: 2M8H[1]). For comparison both structures are shown in front view side to side in (c) and superposed in two orientations in (d). The RBM7 – ZCCHC8 crystal

structure is shown in light green and pink and the RBM7 NMR structure in dark green. The secondary structure elements that are discussed in the main text are labeled and main differences are highlighted with a black circle. (**e**) Zoom-in at the long β2 - β3 loop (residues 40-53), that is similar between the crystal and NMR structure. The RBM7 – ZCCHC8 crystal structure is shown as cartoon and the β2 - β3 loop in the sticks representation. RBM7 is colored green, ZCCHC8 pink. The two Proline residues are highlighted and hydrogens bonds are indicated by lines. (**f**) Comparison of interactions at the back helical surface of RRM domains. The structure of RBM7-ZCCHC8 complex was superposed with eIF3b/eIF3j complex structure[2]. The zoom-in views show how an equivalent hydrophobic pocket in the two RRM-containing proteins can accommodate either a Trp residue in the case of eIF3b – eIF3j or two Leu residues in the case of RBM7 – ZCCHC8. The RRMs of RBM7 and eiF3b are shown in light and dark green respectively. ZCCHC8 is shown in pink and the eIF3j peptide in red. (**g**) Quantitative analysis of RNA-binding properties of $RBM7^{1-98}$ and $RBM7^{1-98}$-$ZCCHC8^{Pro}$ by fluorescence anisotropy using a fluorescein-labeled U_8-mer RNA as a substrate. The data were fitted to an equation describing a single-site binding model to obtain the K_D values. The best fit was plotted as a solid line. The K_{Ds} and their corresponding errors (shown in the inset) are the mean and standard deviation of three independent experiments.

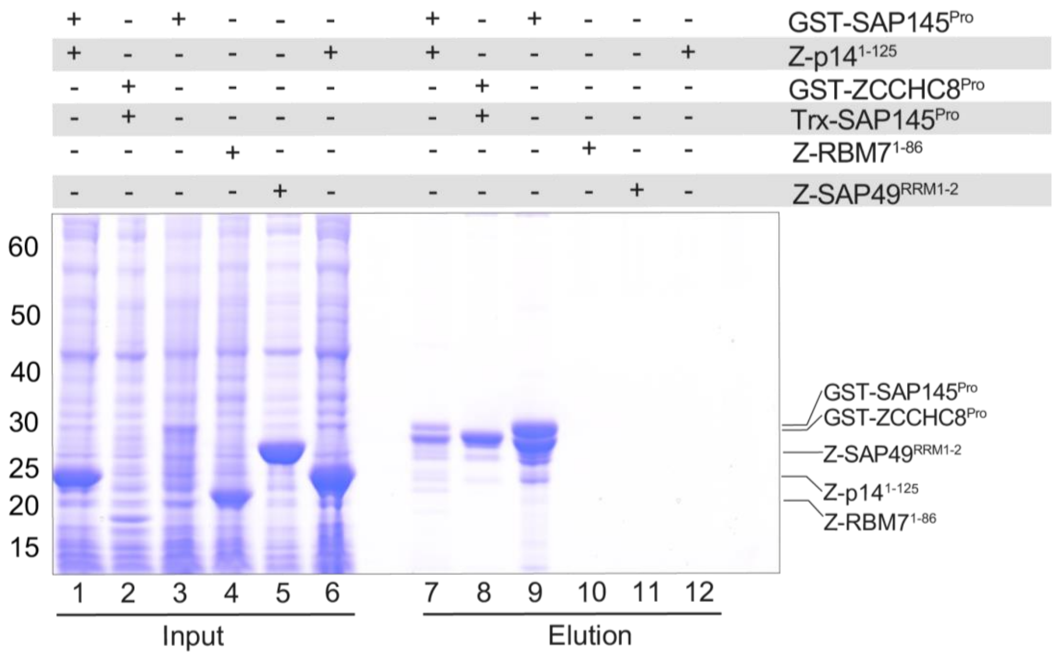

Supplementary Figure 3 SAP145^{Pro} does neither interact with p14 nor with ZCCHC8^{Pro}

GST-tagged SAP145^{Pro} was co-expressed with full-length Z-tagged p14 or GST-ZCCHC8^{Pro}. Pull-down assays were carried out and analyzed as described in Fig. 1B. Neither p14 nor the proline-rich domain of ZCCHC8 interacted with SAP145^{Pro}. As controls the isolated RRM domains of RBM7, SAP49 and p14 were tested for non-specific binding to GSH-resin.

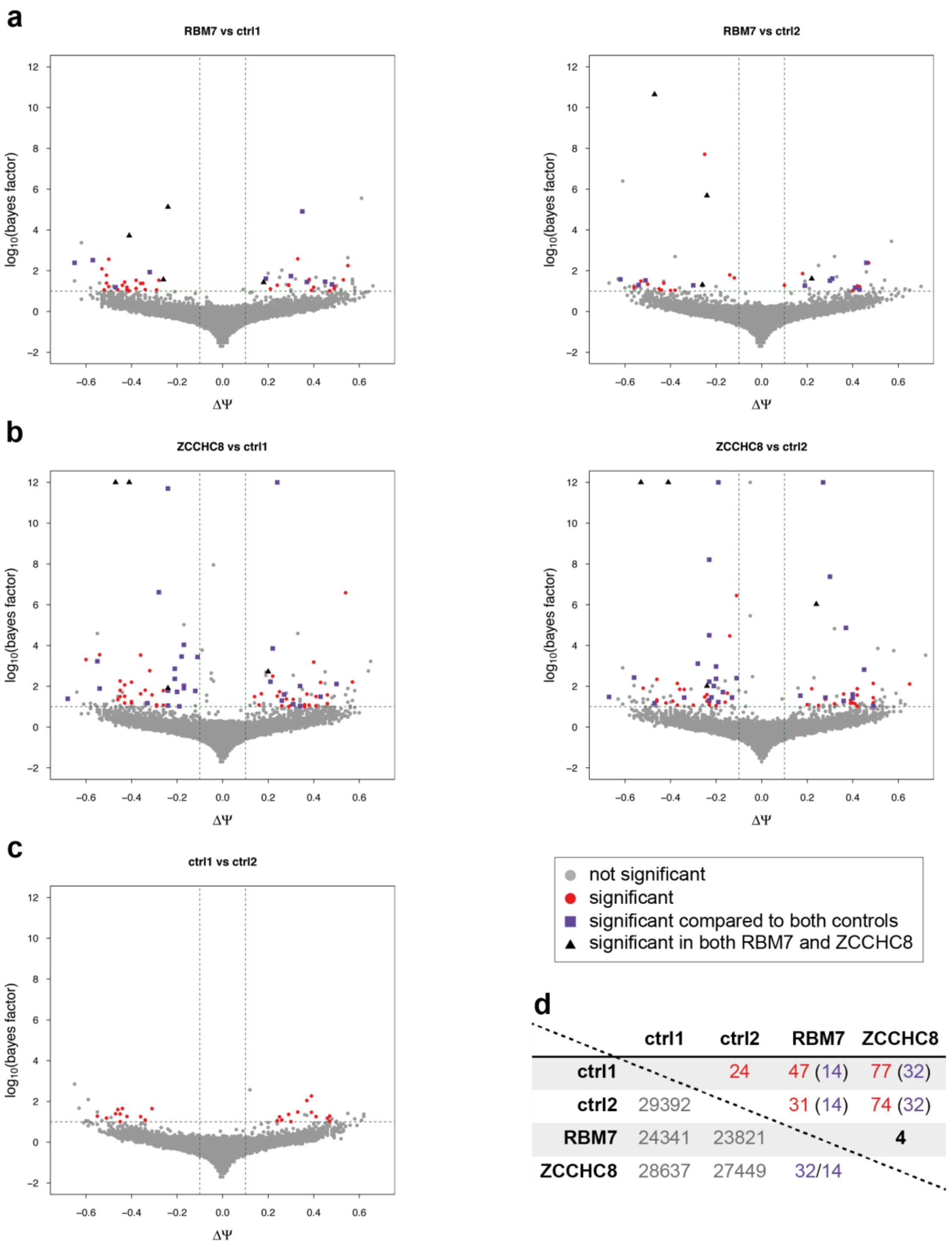

d

	ctrl1	ctrl2	RBM7	ZCCHC8
**ctrl1**		24	47 (14)	77 (32)
**ctrl2**	29392		31 (14)	74 (32)
**RBM7**	24341	23821		**4**
**ZCCHC8**	28637	27449	32/14	

Supplementary Figure 4 RBM7 and ZCCHC8 depletions result in negligible splicing changes

(**a**)-(**c**) Volcano plots of MISO results for all annotated skipped exons, retained introns, mutually exclusive exons, alternative 5' and 3' splice sites. In (**a**) and (**b**), two

different control samples were compared to the RBM7 and ZCCHC8 depletion samples, respectively (left: ctrl1; right: ctrl2). In (**c**), the two control samples were compared. In each individual plot the gray dots indicate alternative splicing events that did not pass the criteria for a significant alteration between the compared conditions (Bayes factor ≥ 10 (indicated by horizontal dashed line); $|\Delta\Psi| \geq 0.10$ (indicated by two vertical dashed lines); Number of reads supporting the first isoform ≥ 1; Number of reads supporting the second isoform ≥ 1; The sum of reads supporting both isoforms $\geq$ 10). Red dots indicate splicing events with significant changes between conditions. Purple squares indicate events that are significant for the given knockdown condition when compared to both control samples (14 for RBM7 and 32 for ZCCHC8), and black triangles show events that are significant in both knockdown conditions when compared to both controls. (**d**) Table showing values related to the plots in (**a**)-(**c**). Above diagonal: the number of significant splicing events in red and the number of significant splicing event compared to both controls in purple and in brackets. Below diagonal: the number of tested splicing events. There are only 4 shared altered splicing events when comparing the 32 and 14 consistent events identified for ZCCHC8 and RBM7 knockdown samples, respectively.

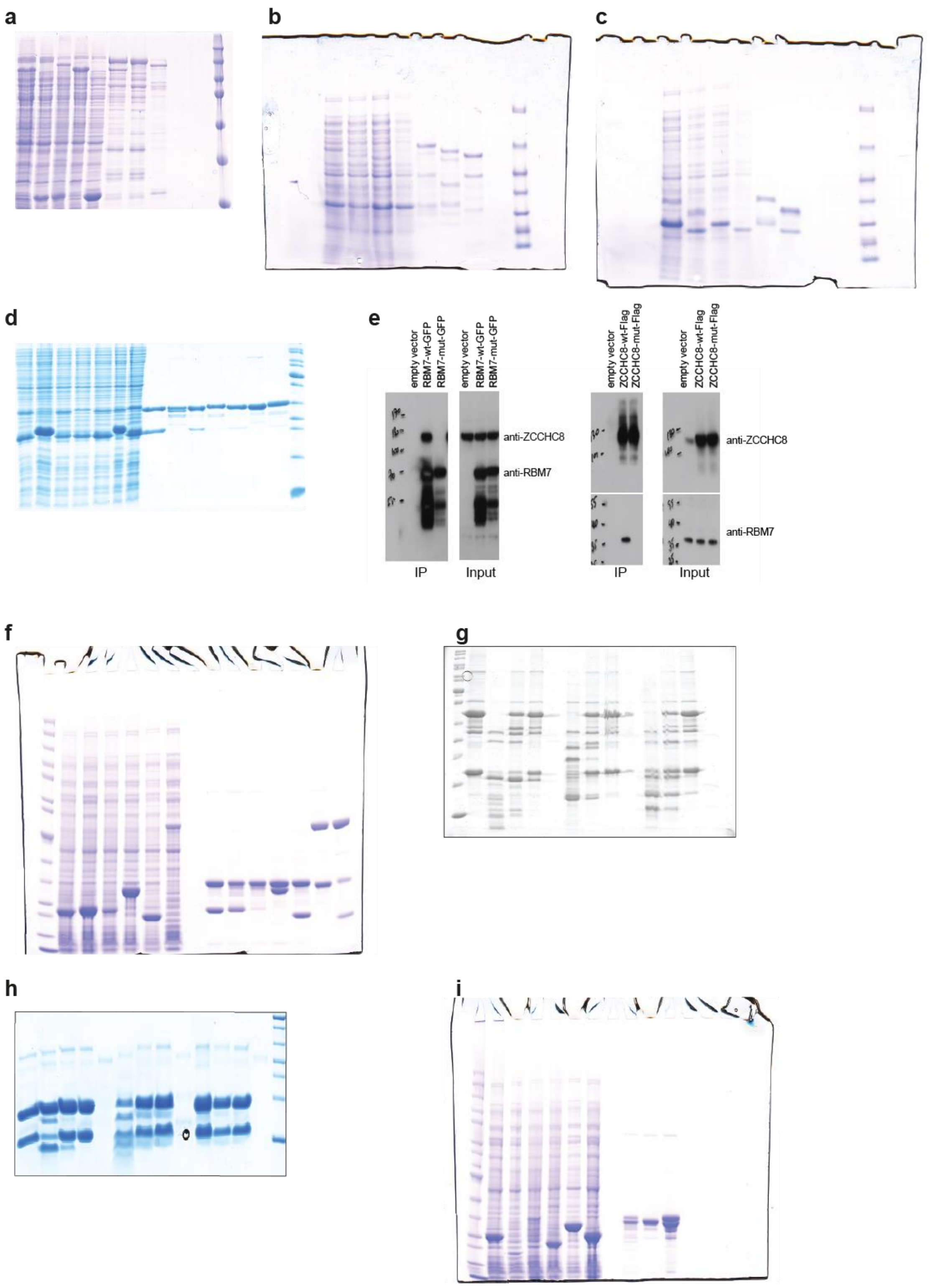

Supplementary Figure 5 Uncropped gels and blots.

(**a**) Fig. 1b, (**b**) Fig. 1c, (**c**) Fig 1d, (**d**) Fig. 3a, (**e**) Fig. 3b, (**f**) Fig. 4b, (**g**) Supplementary Fig. 1a, (**h**) Supplementary Fig. 1b, (**i**) Supplementary Fig. 3

Supplementary References

1. Hrossova, D. *et al.* RBM7 subunit of the NEXT complex binds U-rich sequences and targets 3'-end extended forms of snRNAs. *Nucleic Acids Research* **43,** 4236–4248 (2015).
2. ElAntak, L. *et al.* The indispensable N-terminal half of eIF3j/HCR1 cooperates with its structurally conserved binding partner eIF3b/PRT1-RRM and with eIF1A in stringent AUG selection. *Journal of Molecular Biology* **396,** 1097–1116 (2010).
